# Ensuring Risk Awareness of Vulnerable Patients in the Post-*Montgomery* Era: Treading a Fine Line

**DOI:** 10.1007/s10728-020-00396-9

**Published:** 2020-05-17

**Authors:** Sandip Talukdar

**Affiliations:** 1 School of Law, University of Manchester, Oxford Road, Manchester, UK; 2 Cumbria, Northumberland, Tyne and Wear NHS Foundation Trust, Carleton Clinic, Cumwhinton Drive, Carlisle, CA1 3SX UK

**Keywords:** Informed consent, Decision-making, Vulnerable patients, Montgomery, MCA 2005

## Abstract

The 2015 UK Supreme Court judgment in *Montgomery v Lanarkshire* reinforces the importance of informed consent to medical treatment. This paper suggests that *Montgomery* recognises the challenge faced by vulnerable individuals in choosing between treatment options and making decisions with appreciation of information about material risks. The judgment endorses a form of weak paternalism to safeguard such persons, which is not disrespectful of the aggregate principles of the Mental Capacity Act 2005. But ethical practice requires professionals to tread carefully between weak and hard paternalism in the context of therapeutic interactions with vulnerable patients, while ensuring their awareness of material risks.

## Introduction

The Mental Capacity Act 2005 (MCA) and the judgment of the UK Supreme Court in *Montgomery v Lanarkshire Health Board* [38]^1^ rank among the cornerstones of English medical law. The MCA applies to persons who lack decision-making capacity. *Montgomery* relates to persons of ‘sound mind’, and stipulates that such persons must be informed about material risks of the proposed treatment and available alternative options of intervention [38 at 87]. The judgment includes exceptions for persons unable to consent in a clinical emergency, competent patients ‘waiving’ their right to be informed about risks, and the ‘therapeutic exception’ where doctors may intentionally withhold information to protect patients [38 at 85, 88 and 91]. Failure to take reasonable steps to ensure the patient is aware about material risks is a breach of the duty of care, and would count as negligence when there is resultant harm. This paper will consider the influence of vulnerabilities on decision-making by patients about their treatment; and suggest that in spite of implicitly acknowledging this issue, *Montgomery* has also contributed to newer vulnerabilities. It will be argued that empowering vulnerable patients necessitates helping them to correctly appreciate material risks.^2^ The paper will explore whether such process breaches the MCA.

The paper commences with brief overviews of the concept of vulnerability, the core elements of *Montgomery* and the principles of the MCA. This will be followed by an argument in favour of weak paternalism to limit harm arising from decisions made by vulnerable patients with inadequate awareness of material risks and alternative options of intervention. It will be highlighted that the approach is congruent with the stipulations laid down by *Montgomery.*

Coggon and Miola have stressed the importance of distinguishing between autonomy and liberty in relation to deciding about treatment, and argued that the exercise of reason based on ‘good ground of knowledge and understanding’ is crucial to autonomy in medical law [12, p529]. This Kantian interpretation of autonomy is relevant to the discussions in this paper. Coggon and Miola have also highlighted how English courts have supported the Millian notion of liberty in the desire to promote autonomy [12, p533]. This is important because *Montgomery* has been heralded authors like Badenoch as the triumph of patient autonomy that marks the beginning of the end for medical paternalism [4, p12]. But *Montgomery* has also attracted criticism for its consumer-focussed approach to healthcare that requires patients to make informed choices, which may be challenging for vulnerable individuals [49].

Buetow and Elwyn have argued that patients are morally responsible for decisions about their own treatment [6]. In a similar vein, Draper and Sorrell suggest that being vulnerable does not automatically exclude patients from obligations towards themselves [14, p339]. Such perspectives risk overlooking constraints on autonomy that are induced and perpetuated by vulnerabilities. Holding vulnerable persons responsible for unfavourable outcomes is problematic, and is compounded when professionals overlook vulnerabilities.

## Vulnerability and Post-*Montgomery* Decision-Making

### Concepts of Vulnerability

‘Vulnerability’ is an oft-used but debated term [43, p1058]. Schroeder and Gefenas interpret vulnerability as being ‘defenceless, liable, imperfect, unprepared, frail, susceptible, weak, helpless, open to, exposed to danger’ [53, p114] which resonates with the view of Mackenzie and co-authors that fragility and susceptibility to suffering are key elements of the concept [32, p4]. Martha Fineman provides an ontological interpretation of vulnerability as a ‘universal, inevitable, enduring aspect of human condition’ in arguing that vulnerability originates from our existence as embodied beings, which results in humans being continuously confronted with risks of ‘harm, injury and misfortune’ [19, p8]. The intricate relationship of vulnerability to the human body has been noted by other authors. Bryan Turner associates embodiment with the propensity to sickness, infirmity, suffering and death [58 p29], and Judith Butler argues that vulnerability is inextricably associated with social interaction and interdependency [7, p22].

Fineman also suggests that preoccupation with identity-based issues may cloud scrutiny of the limits of social justice and governmental responsibilities [20, p254]. Goodin perceives vulnerability to be essentially relational in nature [22, p112], and Allen Wood interprets vulnerability as the key to exploitation that facilitates exertion of power by one party over another [59, p142]. Goodin describes vulnerability as a state of being under a perpetual threat of harm [22, p110]. Such threats may affect physical and psychological domains of a person, and extend to economic, institutional and moral harms [43, p1058]. The overall harm may extend beyond the pathological process of an illness, and include all harm resulting from her interactions with healthcare systems and professionals.

There is general agreement on two broad sub-categories of vulnerability, though some authors believe a third category needs to be considered. The first is inherent vulnerability, described by Fineman as vulnerability that results from the embodied existence of human beings, which suggests that some forms of vulnerability are not eliminable [19]. The second is situational vulnerability, which is context-specific and results from social, political, economic, or environmental factors exerting their effects in brief, enduring or intermittent manner on persons. Examples include sudden unemployment, withdrawal of benefits or loss of other social support. Pathogenic vulnerability is said to exist as a ‘subset’ within situational vulnerability but caused by primarily ‘interpersonal’ issues. Goodin describes it as ‘morally unacceptable vulnerabilities and dependencies which we should, but have not yet managed to, eliminate’ [22, p203]. Apart from interpersonal issues like potential or actual abuse, this category would include political or social domination and oppression. Stigma associated with mental disorders is an appropriate example in this regard. Dunn, Clare and Holland suggest that situational vulnerability is more complex than its inherent counterpart and the latter may induce the former—thus causing a ‘vulnerable adult’ to become ‘doubly vulnerable’ [15, p238].

The legal interpretation of vulnerability lays greater stress on the inherent characteristics of the person at risk of experiencing harm [45, p54].^3^ But this needs to be considered with situational issues. An example of the latter is the disadvantageous position of patients during consultations [44] due to imbalance between ‘the knowledge and objectivity of the doctor and the ignorance and subjectivity of the patient’, as described by Lord Templeman in *Sidaway* [54, p904]. Seemingly unwise decisions of the patient coupled with markers of vulnerability could call her decision-making ability into question. In contrast, apparently ‘wise’ decisions (such as when a patient demurely accepts the suggested treatment) may also be associated with risks that she might not have appreciated, but which does not get subjected to similar scrutiny.

Appreciation of risk-related information is addressed in *Montgomery*, and will be discussed later. But at this juncture it is necessary to summarise the main stipulations laid down by the judgement, which need to be read alongside the key elements of the MCA.

## *Montgomery* and the MCA: Key Elements

*Montgomery* centred upon non-provision of risk-related information by an obstetrician to Mrs Nadine Montgomery, who was vulnerable to complications during childbirth on account of her short stature and diabetes. She was not offered a reasonable alternative to vaginal delivery in the form of elective caesarean section. Mrs Montgomery experienced severe problems during her labour, and her child was afflicted with cerebral palsy [38 at 6–22]. The Supreme Court upheld the negligence claim over non-disclosure of material risks and elective caesarean section not being offered an alternative.

The judgment includes three core elements relevant for professionals. First, the ‘materiality’ of a risk depends on whether ‘a reasonable person in the patient’s position’ would be likely to find the risk significant, or if the doctor is (or reasonably should be) aware that a particular patient is likely to find it significant [38 at 87]. Second, the doctor must explain those material risks to the patient in a ‘comprehensible’ [38 at 90] manner in the course of a dialogue [38 at 88] while taking ‘reasonable care to ensure’^4^ that the patient is aware of the material risks and reasonable alternatives to proposed intervention [38 at 87]. Third, the therapeutic exception (when the professional withholds information after concluding that disclosing a particular risk would be seriously detrimental to the patient’s health) is permitted. but it is a limited exception and must not be abused [38 at 90–91].

*Montgomery* refers to persons of ‘sound mind’ who have the right to know about material risks [38 at 87]. It is not clear what ‘sound mind’ denotes in contemporary law and how persons perceived to lack this attribute should be treated in relation to disclosures about risks. One view is that *Montgomery* is applicable to psychiatry patients who possess capacity [27, p98]. But an evolving body of case law suggests that loss of capacity may result from causes other than mental health issues. Munby J observed in *SA* that a person may be ‘incapacitated from making the relevant decision by reason of such things as constraint, coercion, undue influence or other vitiating factors’ [47 at 79]. More recently, the *BF* case re-emphasises that vulnerability may in itself cause a person to be ‘of unsound mind’ even when the MCA test of capacity is satisfied [1].^5^ The case concerned a blind and infirm 97-year-old gentleman who was living in squalor under undue influence of his son. The judge ruled that his vulnerability necessitated the use of Court’s power of inherent jurisdiction, in spite of the gentleman making a capacitous statement of wanting to return home from his care placement [1 at 31–34]. The judgment highlights how vulnerability may induce choices that are detrimental for the individual, and demonstrates the lacuna in law that is being plugged through inherent jurisdiction powers of the court. Interpretation of vulnerability and the perceived need to protect a vulnerable individual are among the key drivers to this situation.

Discussion into whether ‘sound mind’ equates to absence of vulnerability is beyond the scope of this paper. The pertinent issue is that a patient needs to choose between options after being aware about risks and benefits associated with proposed and alternative options of treatment, and vulnerability may influence this process. If a patient is unable to demonstrate this awareness or make an informed decision in spite of practicable help, then questions about her decision-making capacity could arise. The MCA would need to be considered when there is suggestion or evidence of an underlying mental health issue [35, s2–3].

In contrast with *Montgomery*, the MCA provides a legal framework for others to make decisions on behalf of persons who lack decision-making capacity owing to an impairment or disturbance in functioning of the mind or brain [35, s3]. The MCA describes a ‘functional test’ of capacity encompassing abilities to understand and retain relevant information, use or weigh such information as part of the decision-making process, and communicate the decision by any means [35, s3(1)]. Lack of capacity to decide on a specific issue at a particular time must be caused by the said impairment/disturbance of the mind or brain. Establishing this ‘clear causative nexus’ is essential for judging a person to lack capacity [42 at 52].

The above elements of capacity are equally applicable to deciding about medical treatment. However, ‘use of information’ is a broad term and would include instances where a patient might consider and discard a piece of information without appreciating its importance and relevance. This may occur for a variety of reasons including her pre-existing values, beliefs, or undue influence of others. The case *Re T* was an example of the latter, where a critically ill woman refused a blood transfusion, with her decision seemingly being influenced by her mother [48]. The Court of Appeal observed that such circumstances need examination of the ‘scope and basis’ of the decision [48 at 33]. Such examination would involve exploring the patient’s thinking process, her reasoning ability and underlying beliefs about her life, family and society among others.

Doubts over a patient’s processing of information and applying it to herself could precipitate an in-depth assessment of her capacity. This issue will be discussed later, but at this point it is helpful to review the first three principles of the MCA, which mention

1. A person must be assumed to have capacity unless it is established that he lacks capacity [35, s1(2)]
2. A person is not to be treated as unable to make a decision unless all practicable steps to help him to do so have been taken without success [35, s1(3)]
3. A person is not to be treated as unable to make a decision merely because he makes an unwise decision [35, s1(4)].

The fourth and fifth principles refer to persons whose lack of capacity is established, and are not relevant for this discussion.

The Code of Practice to the MCA states that capacity may be assessed when there is ‘doubt’ about the person’s decision-making abilities as a result of her behaviour, circumstances, someone else voicing a concern about her capacity, or when she has already been shown to lack capacity to decide on another issue [13, para 4.35]. Routine assessment of a person’s understanding and interpretation of information in absence of obvious doubt may therefore appear problematic, and requires further elaboration.

It is suggested that the presumption of capacity needs to sit alongside the need to ensure awareness of risks and alternative options of interventions. But awareness cannot occur in absence of understanding. ‘Understanding’ is one of the elements underpinning the definition of capacity by the MCA and is an innate *ability* of the patient. *Montgomery*, on the other hand, refers to the need to ensure awareness, which is a *responsibility* for the healthcare professional [38 at 82 and 87]. But ensuring awareness without errors requires estimation into what the patient may actually have understood, and it is important that it should be devoid of errors. Such estimation would indicate her ability at understanding, since the latter is the first of the abilities required for capacitous decision-making. It is therefore difficult to avoid conflation between an ability of the patient, and the result of her exercising the said ability during instillation of awareness into risks of treatment.

A narrow interpretation of the first principle would additionally contribute to this presumed conflict. It is fair and logical that when a person needs to make a decision on the basis of newly acquired information, she should be able to do so after correct understanding and reasoning that would enable her to apply the information to her own context. If the clinician identifies distinct difficulties with her comprehension or appreciation, then additional measures to aid decision-making would be necessary congruent with the second principle. An estimation of the amount of ‘practicable steps’ to help her make the decision would also be necessary.^6^ In other words, the clinician would need to confirm that the patient has understood and interpreted the information to her individual situation in accordance with her values.

The second principle is of limited utility when clinicians are restricted from confirming the above points. The language of the second principle indicates the Act’s acknowledgement that there are persons who need additional help and support in making their decisions, and which is vital for patients experiencing vulnerability on one or more fronts. Identifying this population would be difficult when there is a bar on exploring how patients may utilise information, and such proscription should be viewed as a systemic contributor to patient vulnerability.

There is no existing suggestion that the first three principles of MCA are hierarchical, or the first principle must be accorded greater importance than the second and the third.^7^

It is actually the second principle that may have greater relevance for persons who have problems with appreciating information, or making a decision congruent with their values. A constricted interpretation of the first principle would entail the professional assuming that the patient has the abilities to understand, retain, and weigh information, and which must be respected at the onset. Such presumptions may lead to the patient’s misperceptions, misinterpretations or uncertainties remaining obscure,^8^ or result in the patient accepting risks of magnitude that she might not have appreciated.

In contrast with manifest vulnerabilities such as speech or communication problems, it is the ‘unseen’ inherent and situational vulnerabilities that might evade attention in absence of efforts to ensure the patient has acquired accurate awareness of risk-related information. This is not to suggest that the second principle be used as a conduit to guarantee the making of ‘good’ decisions that are desirable for doctors. Instead, it highlights the significance of this principle in facilitating choices based on correct interpretation of information in the light of values and circumstances of individual patients.

### Post-*Montgomery*: Recent Vulnerabilities

Situational vulnerability of patients may be perpetuated in healthcare settings by multiple factors. Examples include time constraints, complexities of relevant information and difficulties with comprehension. Interpreting statistical uncertainties may be a challenge for many [28, p802]. Other inherent vulnerabilities such as dyslexia, disorders of attention, or cognitive impairments may hinder a patient’s application of provided information to her circumstances. Miola and Heywood have pointed out that apart from *Montgomery*’s stipulating that patients should not be bombarded with technical information and the information should be ‘comprehensible’ [38 at 90], the judgment provides little guidance about how complex information about treatment and risks should be shared [37].

The importance of correct appreciation of risks cannot be overstated, particularly for patients with ‘unseen’ vulnerabilities. A printed medication information sheet is of little or no utility for a person with dyslexia or someone with limited English language skills. *Montgomery* states that ‘the doctor’s duty of care takes its precise content from the needs, concerns, and circumstances of the individual patient, to the extent they are or ought to be known to the doctor’ [38 at 73]. Failure to identify such needs, concerns, and values may result in doctors adopting defensive measures that may introduce additional vulnerabilities for the patient.

Doctors have expressed concern about how adhering to *Montgomery* may affect their practice [24, 39, 46, 56]. John Reynard provides the example of a rare but profound outcome of a common surgical procedure (Fournier’s gangrene occurring after elective circumcision, with an estimated risk of 1 in 50,000) and his practice in providing a 100-word description about this risk to every patient scheduled for circumcision, specifically mentioning the 30% chance of death if it does materialise [50]. This is a misinterpretation of *Montgomery* since only the ‘material risks’ need to be divulged as opposed to every possible risk. It would appear that doctors may mistakenly equate ‘material risks’ with the possibility of ‘any rare risk materialising’, and which would have the added effect of bombarding the patient with information that can prove confusing or challenging.^9^

Academicians have opined that doctors’ concerns over the *Montgomery* stipulations are ‘overblown’ [16, p124] and good medical practice already requires adoption of the model laid down by the judgment [16, p125]. Such perspectives need to be viewed with the requirement for professionals to designate risks as ‘material’ or ‘not material’, with professional skill and opinion being relevant to the designation [38 at 83]. Doctors may argue that any rare risk with profound consequence such as death needs to be held as ‘material’. This perspective ignores the values of the individual patient from risk categorisation. Whether the patient should be informed about a particular material risk is unrelated to medical knowledge and expertise, and is an issue for the courts according to *Montgomery* [38 at 83]. A particular patient may also contribute to the categorisation of a risk as ‘material’ or ‘not material’, and for this to occur the doctor needs to be aware about her values and preferences^10^: an individual patient may refuse to accept a risk that most patients may not view as problematic, and another patient may willingly accept a risk that others might view as inordinate.

It has been mentioned earlier that *Montgomery* describes two contributors to materiality of risks: whether a ‘reasonable person in the patient’s position’ would find it significant, or whether the doctor is, or reasonably should be aware, that the particular patient would find it significant [38 at 87]. It is not clear as to why a reference to a hypothetical reasonable person is necessary. The judgment in its entirety is centred on the interaction between an individual patient and the doctor, along with stipulations of the latter’s responsibilities. This is one point in the judgment where an external standard is introduced.

‘Reasonable person’ is a legal construct who does not exist in reality [25 at 1–5], and whose knowledge, qualifications, and personal traits are difficult to compare with that of the average citizen [34, p54]. Furthermore, ‘reasonable person’ is not a unitary concept [25]. A disproportionate attention to this legal construct risks shifting attention away from aspects and requisites of the individual patient. The needs of a vulnerable patient are different from that of the hypothetical reasonable person (who may have no particular needs at all). The introduction of the ‘reasonable person’ in the framework thus obfuscates the individual patient-centred approach and can be interpreted as a new vulnerability.

A patient’s comprehension can be influenced by concomitant stress, coping skills, intelligence, and available support systems. She may be overwhelmed by the unexpected responsibility that she is suddenly asked to assume, all squeezed within the limited consultation time. Vulnerability in itself may influence the amount of risk that a patient might find acceptable [40, p2]. A preference for a riskier option, or refusal of an essential intervention may point towards an underlying vulnerability, or if the patient has not acquired sufficient awareness about a particular risk to her situation. A restricted deference to the first principle of MCA would impede doctor’s testing of the patient’s awareness.

It may be said that at its core, *Montgomery* needs an informed patient; who is viewed as an independent and autonomous consumer. But adopting the framework across medical settings ignores the human vulnerabilities associated with ill-health and suffering. Treating vulnerable patients as autonomous consumers risks doctors overlooking, exacerbating or creating new vulnerabilities.^11^ Avoidance of additional harm is among the basic principles of medicine, and ethical practice would necessitate ensuring the patient has acquired adequate awareness about material risks. This is discussed further in the next section.

## Ensuring Awareness

It will be argued in this section that in order to ensure ‘awareness’, confirmation of simple or basic ‘understanding’ is inadequate, and requires evaluation of the patient’s actual appreciation of risks.

*Montgomer*y does not elaborate on how doctors should ‘ensure’ that the patient has acquired awareness of risk-related information, but some form of confirmation would appear a logical necessity. Requesting the patient to provide a gist or summary of the information back to the doctor would count as one option. If there is any error on the patient’s part, then further reiteration of the information may be necessary, and additional comprehension aids may require consideration. It was noted in *Chatterton v Gerson* [10] that a patient needs to be ‘informed in broad terms of the nature of the procedure’ for her to provide legally valid consent to treatment, [10 at 433], which is the distinction between trespass or battery and negligence in English medical law. Given its relevance to consent, his should be the minimum base standard for information about material risks as well.

There is scope for debate as to whether probing the patient’s understanding and attempts at reinforcement of information are paternalistic, particularly if the patient’s response subsequently leads to formal examination of her decision-making capacity. The crux of the issue lies in the interpretation of ‘awareness’.

It is difficult to come across a legal definition of ‘awareness’ in English healthcare law. General interpretation of being ‘aware’ conveys being informed, cognisant, conscious, and sensible [41], though questions remain over the origin of the concept and how it may be measured [11]. There is agreement that ‘awareness’ in situations that involve decision-making calls for a level of exertion of cognitive faculty that is over and above what guides mundane behaviour [30, p425].

Quoting multiple authors, Margaret Yaruke identifies three levels of awareness, where the basic level involves ‘mere apprehension of an object without active attention’ [60, p260], and where the subject (patient) notices the object but is not obliged to change [60, p266]. The next level of awareness involves development of knowledge based on the acquired senses or evidence that go beyond minimal registration, when the subject is able to interpret a relationship with the object. Yaruke identifies a third level of awareness where the subject develops intellectual recognition of the action necessary on her part, with assumption of responsibility [60, p267]. Similar hierarchical levels of awareness for other complex cognitive functions such as second language learning have been identified by other authors [3, 52 p131–133].

It is suggested that the third level of awareness (as described by Yaruke) is necessary for healthcare-related decision-making, this is because a competent patient needs to be aware of what she might be agreeing to, and the implications it would have for her. Both of these denote an assumption of responsibility attuned to this level of awareness. Coggon and Miola have observed that ‘being informed’ goes beyond simple exposure to information, and requires the patient to ‘comprehend and compute’ the provided information [12, p546]. The doctor’s responsibility for ensuring awareness of material risks therefore extends to scrutiny of the extent to which the patient may have appreciated the information and applied it to her situation. This would be a confirmation of the ‘comprehension and computation of information’ as mentioned by Coggon and Miola.

But there may be instances where this approach extends to exploration of the patient’s reasoning at a level that questions her abilities as an autonomous individual who is capable of rational decision-making. There is thus a fine line between ‘weak paternalism’ in order to promote awareness as a pathway to authentic decision-making, and a ‘harder’ paternalism of probing into capacity at length.

## Weak Paternalism

Beauchamp defines ‘paternalism’ as ‘intentional overriding of one person’s autonomous choices or actions by another person, where the person who overrides justifies the action by appeal to the goal of benefitting, or of preventing or mitigating harm to the person whose choices or desires are overridden’ [5, p81]. Feinberg describes weak paternalism as permissible limitation of autonomy when the paternalistic actor rightfully acts to ‘prevent self-regarding conduct only when it is substantially non-voluntary or when temporary intervention is necessary to establish when it is voluntary or not’ [17, p113]. Sjostrand and others argue that interventions to ensure a patient’s choice to be based on correct appreciation of information are justified and would count as weak paternalism [55], and these may be termed ‘paternalism for the sake of authenticity’ [55, p715–716].

It is possible for a decision to be voluntary without being authentic, as may occur when a patient decides in favour or against a suggested procedure without correct interpretation of provided information. *Al-Hamwi* [2] is a vivid example in this regard and reinforces the observation made by Sjostrand et al. [55]. Mrs Al-Hamwi, whose first language was not English, was told about the risks of amniocentesis during her pregnancy. She misinterpreted the information and believed that the risk of miscarriage with amniocentesis was 75%, whereas it was only 1% in reality. She declined the procedure, and her son was later born with disabilities that amniocentesis would have detected. The judgment observed that clinicians have a responsibility to take ‘reasonable and appropriate steps to satisfy themselves that the patient has understood the information which has been provided; but the obligation does not extend to ensuring that the patient has understood’ it [2 at 69].

The observation in *Al-Hamwi* appears to suggest that misunderstandings and miscommunications are unavoidable in doctor-patient interactions [36, p111]. Assumption that the patient possesses the ability to understand does not mean the patient has indeed understood the provided information [12, p541].^12^ Alisdair Maclean suggests that unquestioned assumptions and ‘non-directive counselling’ imposes ‘the libertarian ideology of mandatory self-determination’ on the patient.[33, p336]. This shortcoming has been addressed through *Montgomery*’s implicit overruling of *Al-Hamwi*,^13^ but the latter case highlights how vulnerability may be induced through assumptions on the part of the professional, and whose likelihood may be increased when the patient makes a choice that is ‘unremarkable’ [12, p545], or does not markedly stand out as unusual.

If anything, it is such ‘unremarkable’ choices (as opposed to seemingly irrational or bizarre choices) that might eclipse underlying vulnerability in absence of efforts on the professional’s part. Weak paternalistic measures would address such predicament and ensure the patient’s decision is based on correct comprehension, which is precisely the recommendation in *Montgomery* [38 at 82] As Coggon and Miola have pointed out, Mrs Al-Hamwi had ‘lost, or never realised, her autonomy’ [12, p539] which would not have been the case if her particular needs had been recognised and addressed. Ensuring accurate awareness of risks can therefore be viewed as autonomy-promoting for patients who are vulnerable for one or more reason. Although the process may necessitate measures in the realm of weak paternalism, it is important that weak paternalism should not be used to guide patients towards ‘good’ or ‘desirable’ decisions; such action would fall in the domain of hard paternalism.

It may be questioned as to what conduct or expectation on part of the patient is being prevented by active efforts at ensuring awareness for it to be viewed as paternalistic. A hypothetical examination of an alternative of *Al-Hamwi* is helpful in answering this question. Had Mrs Al-Hamwi’s comprehension been put to examination, it would have involved challenging her comprehension of the risk of amniocentesis. She had already decided, on the basis of reasoning (but based upon misperception), that it was not a risk worth accepting. Questioning her comprehension would have thwarted her desire to leave the matter at that point with no further consideration of the procedure. This would be paternalistic since it would intervene with what she had decided after logical reasoning, albeit on the basis of erroneous comprehension. In situations as these, explanation of the purpose of further questioning may lead to reengagement of the patient, and the intervention would cease to be paternalistic at that point. But while she lacks the knowledge into why she must revisit the issue, the intervention would retain paternalistic credentials.

Jason Hanna has observed that there is a misconception that weak paternalism permits only temporary interventions to improve awareness [23, p423]. Feinberg believed that if a wrong impression is held over a long time, then sustained intervention is permissible until the person becomes acquainted with the truth [18, p127–128 and 130–131]. It is difficult to assert that such drawn-out interventions over unspecified duration do not violate autonomy, particularly when the rationality of the patient’s choice is put to test. But this might be unavoidable in some situations involving vulnerable patients.

There is thus a risk of overlap between active measures to aid awareness, and assessment into whether the patient’s decision is indeed based on comprehension and retention of information with subsequent ‘weighing or balancing’, since both involve similar cognitive domains. It may also be argued that assessment of the patient’s comprehension and reasoning as a prelude to treatment would be the beginning of a slippery slope leading to further questions over decision-making with an option of recourse to the therapeutic exception [8, p140]. Another pertinent question is whether it is possible to demarcate between purported ‘weak’ paternalism and ‘hard’ alternatives. Telling a patient who prefers not to know about risks that it is an unacceptable way to decide about treatment would be a ‘hard’ approach, coupled with increased influencing of patient choice from ‘persuasion’ to ‘coercion’ [57, p118–119].

A conflict with the ruling in *Wandsworth* [31] may arise if patients are unaware of the purpose of explanation and questioning into their awareness of the risks. Hayden J observed in *Wandsworth* that explanation of the ‘purpose and extent’ of assessment is a mandatory prerequisite to any assessment of decision-making capacity [31 at 49]. Exploration into the patient’s awareness of material risks without explanation of the underlying reason may breach this case law, since both circumstances involve probing similar attributes. But from an ethical perspective, active confirmation of a patient’s awareness with the aim of preventing harm would remain within the spectrum of paternalistic intervention.

The above needs to be viewed with findings that multiple instances of weak paternalism are prevalent in healthcare [9, 29, 51]. *Montgomery* stresses the necessity for clinicians to respect the patient’s ‘entitlement to decide on the risks to her health that she is willing to ‘run’ [38 at 83], which is respectful of autonomy. But the stipulation that professionals should make reasonable efforts to ensure the patient is ‘aware’ about material risks [38 at 87] can be interpreted as a protection for vulnerable individuals against exposing themselves to unappreciated levels of risk. Weak paternalism is thus integral to *Montgomery*, with its implicit recognition of the necessity for correct awareness about material risks that may prove to be the difference between an uneventful cure and an unfortunate outcome.

## Conclusion

This paper has looked into the interaction of patient vulnerability with decision-making in the context of medical treatment. It has been highlighted how vulnerable patients might be at distinct risk when there is inadequate exploration into their awareness of risks, and the safeguard that *Montgomery* provides in this regard. Development of such awareness demands a high level of engagement with the information and cognitive processing, which may be influenced by inherent and situational vulnerabilities. It is suggested that healthcare professionals have a duty to identify vulnerabilities of patients, which includes assessing the patient’s awareness and appreciation of information. The approach is congruent with *Montgomery* and recommendations by the General Medical Council to doctors [21, paras 13–15]; and requires focus on the ‘particular’ vulnerable patient rather than the ‘reasonable person in the patient’s position’.

Compliance with the stipulation laid down by *Montgomery* to ensure the patient is aware of the material risks of treatment can be seen to involve weak paternalism, while being supportive of vulnerable patients in making authentic choices about their treatment. Not questioning or confirming a patient’s decision on the ground that it would be an assault on her autonomy could subsequently expose her to greater harm for which the doctor would bear moral responsibility. However, there is a clear need for professionals to tread carefully between weak and hard paternalism. It is also a legal necessity to keep patients informed about measures to help optimise her awareness about risks and benefits of treatment.

Herring and Wall have warned that having ‘others harm you and to be told no protection is offered because you have chosen this harm, even though it is against your deepest values, is horrific’ [26, p713]. This is pertinent for patients who accept interventions without full appreciation of associated risks, and when such choices are interpreted by others as voluntary decisions without recognition of various vulnerabilities that patients might be subject to. Active efforts at ensuring comprehension and appreciation of information therefore needs to be integrated as an essential aspect of professional practice in healthcare.

## Footnotes

1 The other major statute is the Mental Health Act 1983, but is outside the purview of this article.

2 Provided there is no irremediable issue with the patient’s comprehension.

3 In the context of healthcare, such harm may involve physical, mental or social domains, or a combination of these.

4 Italics added.

5 The MCA test of capacity will be described later in this section.

6 Examples would include providing the information in writing or securing the services of an interpreter, depending on the specific identified needs of the patient.

7 The first three principles must be followed ahead of the principles 4 and 5, which deal with decision-making by others in P’s best interests.

8 As was seen in *Al-Hamwi,* discussed later in this paper.

9 An anxious patient may lay greater stress on the ‘30% chance of death’ on materialisation of Fournier’s gangrene in the example provided by Reynard; notwithstanding the fact that the actual risk of death would be 30% of 1 in 50,000, which equates to 0.0006%.

10 These elements should come to light during the dialogue between the patient and the doctor.

11 I am thankful to one of the anonymous reviewers for highlighting this point.

12 Coggon and Miola highlighted this issue in 2011, 4 years before *Montgomery* laid down the stipulation for doctors to ensure awareness about risks.

13 As pointed out by Miola and Heywood [37].
